# Massage—Its Application

**Published:** 1883-07

**Authors:** Franklin H. Martin

**Affiliations:** Assistant to Nervous Department of St. Joseph’s Hospital, and Clinical Lecturer in the Gynecological Department of South Side Dispensary, Chicago; 2139 Wabash Ave.


### ARTICLE IV.

MASSAGE—ITS APPLICATION. By FRANKLIN H. MARTIN, M.D.,
Assistant to Nervous Department of St. Joseph's Hospital, and Clinical Lecturer in the Gynecological Department of South Side Dispensary, Chicago.

The word *massage*, according to Piorry, is taken from the Greek (rub or knead), and according to Savary from the Arabic word *mass* (softly squeeze).

Massage was practiced by the Chinese over 3,000 years B. C.; it was also practiced with considerable success and favor by the ancient Greeks and Romans. In tracing the interesting and eventful history of this subject, one finds that "movement" treatment, in some form or other, has been practiced by almost every people who have left a medical history. It was practiced rationally by the ancient Greeks and Romans; it was practiced by the ancient Persians, and others of later date, for the superhuman healing powers supposed to be imparted by the hands of the operator; it was and is practiced by the Sandwich and other Pacific islanders as an after-dinner luxury; it has been, and is yet, even in our own present enlightenment, unscientifically practiced by numerous indiscriminating impostors and quacks. On account of its many mysterious and unsavory connections, massage is naturally looked upon by investigating therapeutists with not a little distrust, and in consequence, a fertile field of therapeutics has, until very recently, been left comparatively unexplored. Another reason that may be given for the unpopularity and primitiveness of the therapeutics of massage, is the fact that so many of our general practitioners have not the time to spare from a busy family practice that is necessary to perfect themselves in an art that requires no little practice and knack, as well as a peculiar physiological and anatomical knowledge.

Massage, as now generally accepted by the profession, includes all kinds of mechanical manipulations and movements, of whatever nature, used by the physicians for their curative effect on diseases. Strictly speaking, however, it includes only those kneadings, rubbings, slappings and frictions that can be accomplished by the operative's hands alone, while the patient's body and muscles are allowed to remain practically passive. We, therefore, it will be seen, do not include under the head of massage gymnastic exercises and the so-called "movement cure," although we recognize in them powerful aids in the treatment of many forms of diseases, when discriminately and scientifically applied.

The room in which massage is given should be well carpeted and have connected with it a small dressing and toilet apartment. The temperature of the room should be about 75° F. The patient to be treated, after disrobing, which is absolutely necessary, should lie upon a table or lounge, the person being protected by a light flannel spread. In the office may be used a narrow, upholstered table, without springs, about thirty inches in height, twenty-eight inches in width and six feet in length. It should be well upholstered with moss or hair, covered firmly with rep or leather. In this you have a comfortable, yet, what is desirable, a firm, unyielding bed, of a convenient height. I use for this purpose a table of my own device, the bed of which is in two parts, connected near the center by double-acting hinges, which allow either the head or the foot of the bed to be raised or lowered and retained at any angle. The movement is accomplished by a suitable ratchet combination. The two parts, by a simple arrangement, are made to fold together, when not in use as a bed, making an excellent gynæcological table, and at the same time occupying but little space.

The different movements used by masseurs are many. These movements have been variously named and classified, according to the fancies of different operators. I will adopt the classification used by Professor von Mosengeil in his description of the French method of massage. He describes the movements under four heads: First, *Effleurage;* second, *Massage á friction;* third, *Petressage;* and fourth, *Tappotement.*

The patient to be manipulated should be instructed to lie perfectly passive, with muscles relaxed. When the growth of hair on some particular part to be manipulated is heavy, the surface should be shaved, otherwise, even when the massage is light, inflammation of the hair bulbs is often produced. A thin growth of fine hair is not objectionable. It is not only unnecessary to use a thick oil or lubricating substance on the part to be masséed, as some masseurs recommend, but it positively defeats to a marked degree the principal effects desired. Where a part is lubricated, friction is lost, and friction is highly necessary to complete massage. In fact, lubricants, to my mind hamper all the principal movements by making the parts uncomfortable. However, in some few cases where the skin after the massage is left dry and harsh, you may anoint the surface with a little vaseline, rubbing it in well; this leaves the skin soft, and at the same time obviates any tendency to inflammation of the hair bulbs. In doing away with lubricants, of course we do not lose sight of the fact that it is, many times, desirable to administer certain remedies by inunction. That may be done, however, without reference to massage.

By *Effleurage* we mean centripetal strokes, which ar performed with the palms of both hands. It is the variety of massage that is used in the stroking treatment of inflamed and enlarged joints, œdematous swellings, etc. It is usually necessary to begin with light strokes, until the tender tissues become gradually more and more tolerant, when the pressure of the hands can be increased and the stroke quickened. The increased force should be exerted gradually from the beginning of the stroke, the intensity of the pressure reaching its climax at the middle of the movement and then again lessening, allowing the hand to be removed by gradual lightened pressure while the other hand is being applied. In this way a much greater pressure and more friction is agreeably tolerated than could possibly be if the hands were applied with the same force suddenly. In manipulating an inflamed joint, or a diseased part of any kind, the change of hands should be made at the most painful point, for it is at that point the stroke is lightest. If the part to be operated upon is too small to admit of using the whole palmar surface of the hand, use only the palmar surface of the fingers. The gentle pressure centrally directed. not the friction, is the principal factor in effleurage. Therefore the movements need not necessarily be rapidly performed. A masseur must be well practiced, and at the same time very skillful, to be able to manipulate properly an inflamed knee-joint and use over 150 strokes a minute. It can be done, however, and one can only imagine the great skill it must require, when it is remembered the sensation to be experienced by the possessor of that sensitive knee-joint, is one of agreeable constant pressure.

By these movements, the absorption of pathological fluid exudates is promoted. Exudates in serous cavities, joint cavities, and in cellular tissues, in all parts of the body, are by direct mechanical force dispersed. Venous and lymphatic circulations are primarily directly forced into action, thereby aiding secondarily, by a *vis á frontis*, the arterial circulation. As the circulation is increased, the tissue change is increased, oxidation takes place more rapidly, and thereby the temperature is increased in the part. It can readily be seen without much stretch of the imagination, how absorption of extravasated blood, blood exudates, or the products of old chronic inflammation, is promoted by effleurage. By the strokes with accompanying pressure, the pathological fluid is spread over a greater space, allowing it to come in contact with a greater number of absorbents, the more solid products of disease are emulsified, and the solid particles subdivided, allowing them to be more readily taken up by the absorbents. The absorbent vessels, both capillary, vascular and lymphatic, are capable of more work, on account of the tonicity given them by the increased blood supply, and therefore absorb with greater rapidity the foreign particles of tissue that have already been partly prepared for absorption. Not only does all this seem rational, but still more, the stroking movements in the direction of the venous and lymphatic vessels, with the accompanying pressure, not only naturally, but actually forces, by mechanical pressure, the flow of their contents. Some therapeutists go so far as to say that the emulsified particles are actually pressed into the absorbent vessels. Stroking in any other direction than toward the blood center is only applicable in exceptional cases, on account of its directly retarding the lymph and venous circulation. Still, there are cases, for instance, where there is an extraordinary fluid accumulation, in which stroking in different directions might assist in spreading the fluid over a greater absorptive territory, and thereby facilitate the absorptive process.

Every inflammatory swelling, of course, it would not be safe to treat by massage, as the tissues might contain infectious material, and by promoting its absorption or distribution, the infection would contaminate otherwise healthy tissues. Massage is absolutely contraindicated in all cases of venous inflammation, on account of the liability of emboli being detached and thrown into the circulating blood from the diseased coats of the vessels. The evil consequences of such a result can easily be imagined. The absorption of soft fibrous tissues, fungoid accumulations, etc., may be promoted by the crushing that their tissues receive by the proper application of effleurage.

After this stroking process has been performed for some time, the skin becomes red, the patient feels a warm glow over the surface of the part, and by the touch, as well as the thermometer, the temperature is found to be increased. This condition of stimulation lasts for some hours after the manipulation has ceased, and a delicious sense of restfulness in the part is experienced by the patient as the result.

Where the parts to be manipulated are very tender, effleurage should be commenced by using light and not very energetic strokes over the diseased portion, until the over-sensibility gradually diminishes.

*Massage á friction* is the variety of manipulation that is accomplished by the finger-tips of one hand vigorously rubbing from the periphery toward the center, followed by the finger-tips of the other hand with a slow, stroking movement. This variety is used both in general massage, and also in promoting absorption of superficial inflammatory exudation. The portions of semi-solid morbid tissue that the finger-tips of the first hand crush and rub up or emulsify, the other hand spread and determine toward the center, and thereby promote their absorption. The proper performance of massage á friction requires more skill and patient practice than any of the other varieties of massage, because of the entirely different movements required of the fingers of the two hands. It is peculiarly difficult to perform the rapid rubbing movements with the left hand while the right follows with the slow stroking movements.

By *Petrissage*, we mean a kneading of the parts. One grasps deeply a fleshy mass of tissue, and rolls it gently in the hands, allowing it to gradually escape from one hand as it is grasped with the other. As the part is being rolled in the hand it should be squeezed with more or less force, according to the toleration of the tissues. The general rolling movement should be in the direction of the center of the body. Where the muscles of a limb are receiving this treatment, the hands should grasp the limb on either side at the distal end, and an irregular, rolling motion of both hands in opposite directions, with considerable pressure, and a constant determination of the movements toward the central end of the limb, the hands not being removed until the whole limb has been traversed.

Petrissage is used in kneading the deep muscles of the back and the different viscera that can be reached through the thin abdominal walls, as well as the muscles of the abdomen themselves. Through the walls of the abdomen the uterus, the liver and the spleen can with greater or less success, according to the corpulency of the patient, be manipulated by this method, while the stomach, the intestines, especially the large intestines, can be influenced by this kneading process as by no other method.

*Tappotement* is the name of the last division of massage that we have to describe. It includes all the flagellations, the slappings, beating and pounding movements that may be performed with the hands of the operator, or any little device he may find to take the place of the hand. Tappotement may be performed with the flat palm of the fingers, by a succession of light, rapid slaps, or the whole open palm of the hands by a succession of slower, harder blows. The effect of the former is simply to stimulate the circulation in the skin; the latter affects in the same way somewhat deeper tissues. The tips of the fingers, the ends on a plane, may be used; the ulnar border of the hand is often used; where harder blows are required, the knuckles of the closed hand may be used; there are also various kinds of instruments that have been manufactured for this purpose, from India-rubber, whalebone, wood, and other materials. These devices serve some very good purposes, but cannot compare in efficacy with the well-trained hand.

General massage includes all these varieties of manipulations, systematically combined and rationally applied, that we have with considerable minuteness described. In applying massage for its general effect on the system, the masseur should commence, 1st, at the hands, and manipulate in the direction of the trunk; 2d, with the feet, and manipulate in the direction of the trunk; 3rd, with the head; and 4th, with the trunk itself, directing all manipulations toward the center of circulation.

To commence, the manipulator takes the distended hand of the patient in his left hand with either the palmar or dorsal surface uppermost, and supporting it in that position, applies friction by rapid ascending strokes with the palmar tips of the fingers of the right hand. These movements are applied to the palmar dorsal and sides of each and all the fingers; they are then each rolled transversely in the closed hand of the operator.

The same ascending rapid friction strokes will then be applied to the metacarpal and carpal region with one hand, while the other may follow with slower strokes, in the same direction; these movements to be followed by gentle kneading of the more fleshy parts. Passing on to the forearm and arm, similar movements may be repeated (from the wrist to the elbow, and from elbow to shoulder) to those used in the metacarpal and carpal regions—that is, massage á friction followed by pettrisage. The arm is a very convenient portion of the body for both of these processes. The strokes necessary are of convenient length, and the fleshy masses are easily kneaded. About five minutes should be occupied in thoroughly manipulating each upper extremity.

The feet may be manipulated in the same general manner as the hands, the strokes, of course, necessarily being longer. The metacarpal and carpal region are first treated with ascending strokes in rapid succession, followed by the slower kneading strokes.

Transverse kneading is applied by grasping the foot in both hands, and drawing and rubbing the tissues in either direction. The leg and thigh come next in succession; each in their turn are treated similar to the corresponding parts of the upper extremity. More time will necessarily be required, however, in manipulating the large, unwieldy muscles of the leg and thigh than the corresponding parts of the upper extremity. In kneading the fleshy masses at the upper part of the thigh and gluteal region, commence kneading the superficial muscles with the fingers, and as the deeper muscles are called in, use both hands with considerable pressure, and impart to the mass a rolling motion, endeavoring to have the direction of the motion in opposite directions. The tissues of the back are next manipulated. A convenient place for commencing is at the back of the neck, beginning with descending strokes from the occiput. Every portion of the back should first be gone over by the massage á friction, vigorously applied. The strokes here may be both ascending and descending, and of a length convenient to the operator. These rapid, short strokes of one hand, followed by the slow, regular strokes of the other, are to be followed by effleurage and deep pettrisage. The latter should be given special attention, as it is the only method by which the deeper muscles of the back can be reached. The kneadings here are usually applied transversely, in a direction away from the spine.

The chest is treated in the same general way as the back. The strokes should be from the sternum toward the spine, following the direction of the pectoral muscles toward their insertion. Rapid and short strokings are especially applicable here, followed by long kneading strokes.

The abdomen requires careful treatment. The massage here should be of a slow kneading variety (pettrisage), and the direction that of the ascending, transverse, and descending colon.

After the whole body has been manipulated in the general methods described above, about five minutes should be spent in going over the whole surface with rapid slapping (tapottement) movements, applied by the palms of the hands. These movements may be accomplished with great rapidity by skilful alternating of the two hands.

For the proper application of general massage, about one hour in time is required. The time required on the several parts may be roughly estimated as follows: Upper extremities, each five minutes; lower extremities, each eight to ten minutes; trunk, twenty-five to thirty minutes; and the general tapottement five to ten minutes. Of course, it is unnecessary to say these are not invariable rules. One patient may tolerate and require double the massage of his neighbor. Also, as therapeutical indications vary, so will the intelligent masseur find it necessary to constantly modify his manipulations to meet each individual case.

We find it necessary to postpone for a later writing the consideration of the physiological effects and therapeutical indications and applications of massage, at which time, also, the mode of its application to special organs will be considered.

2139 Wabash Ave.

